# 3D microfluidic liver cultures as a physiological preclinical tool for hepatitis B virus infection

**DOI:** 10.1038/s41467-018-02969-8

**Published:** 2018-02-14

**Authors:** A. M. Ortega-Prieto, J. K. Skelton, S. N. Wai, E. Large, M. Lussignol, G. Vizcay-Barrena, D. Hughes, R. A. Fleck, M. Thursz, M. T. Catanese, M. Dorner

**Affiliations:** 1 Section of Virology, Department of Medicine, Imperial College London, London, W2 1PG UK; 2 Section of Hepatology, Department of Medicine, Imperial College London, London, W2 1NY UK; 3 CN Bio Innovations Ltd, Welwyn Garden City, AL7 3AX UK; 4 Department of Infectious Diseases, King’s College London, London, WC2R 2LS UK; 5 Centre For Ultrastructural Imaging, King’s College London, London, WC2R 2LS UK

## Abstract

With more than 240 million people infected, hepatitis B virus (HBV) is a major health concern. The inability to mimic the complexity of the liver using cell lines and regular primary human hepatocyte (PHH) cultures pose significant limitations for studying host/pathogen interactions. Here, we describe a 3D microfluidic PHH system permissive to HBV infection, which can be maintained for at least 40 days. This system enables the recapitulation of all steps of the HBV life cycle, including the replication of patient-derived HBV and the maintenance of HBV cccDNA. We show that innate immune and cytokine responses following infection with HBV mimic those observed in HBV-infected patients, thus allowing the dissection of pathways important for immune evasion and validation of biomarkers. Additionally, we demonstrate that the co-culture of PHH with other non-parenchymal cells enables the identification of the cellular origin of immune effectors, thus providing a valuable preclinical platform for HBV research.

## Abstract (web summary)

Long-term in vitro models for hepatitis B virus (HBV) infection are important to understand this infection, but are lacking. Here the authors develop a microfluidic primary human hepatocyte organoid culture system that can be maintained over 40 days and recapitulates all of the steps of the HBV life cycle.

## Introduction

Globally, more than 240 million people are chronically infected with hepatitis B virus (HBV)^1^. HBV infection is associated with the development of liver cirrhosis and hepatocellular carcinoma. HBV belongs to the family of *Hepadnaviridae* and comprises of a small (3.2 kb), partially double-stranded DNA genome, which persists as covalently closed, circular (ccc)DNA episome. This cccDNA is the transcriptional template for pregenomic (pg)RNA and subgenomic (sg)RNA species. HBV genome amplification includes the reverse transcription of pgRNA within assembling progeny virions^2^. This step is the target of the majority of drugs currently in clinical practice, but since this represents a late stage during infection, treatment with nucleo(s)tide analogs including lamivudine, entecavir, or tenofovir do not result in the elimination of cccDNA. Studying the life cycle of HBV has been complicated mostly due to the lack of suitable in vitro models recapitulating all steps of the viral life cycle in hepatocytes and the difficulty of modeling physiologically intact host cells for HBV replication.

Hepatocytes, which are the target cell for HBV, form complex three-dimensional (3D) structures in vivo, lining the hepatic sinusoids^3^. Despite the ability to isolate viable primary human hepatocyte (PHH) from liver resections, the in vitro cultures remain challenging, mainly due to their de-differentiation as evidenced by their loss of cytochrome P450 (Cyp450) activity and morphological changes within days of culture^4–6^. This has led to the development of several culture models, which better support hepatocyte differentiation, notably the use of fetal hepatoblast cultures^7^ as well as the micropatterning of PHH and non-hepatic stromal cells^8^. Although these culture models recapitulate certain aspects of HBV infection, they are not susceptible to patient-derived HBV isolates, only supporting transiently low levels of infection or require immunomodulation in order to stably replicate HBV^8^. Other model systems, including single-channel microfluidic hepatocyte cultures^9^ and organoid cultures^10^ are promising in their development, though they have not been evaluated regarding their susceptibility to HBV. Additionally, they do not fulfill the requirement for multiplexing, which is essential for preclinical evaluation. Notably, very high viral titers are required to initiate infection in most culture systems, often necessitating multiplicities of infection (MOIs) of up to 4 × 10^4^ HBV genome equivalents (GE)/cell, the presence of dimethyl sulfoxide (DMSO) and polyethylene glycol (PEG) to enhance virus binding^11^, or the use of immunomodulators including Janus-activated kinase/signal transducer and activator of transcription factor (STAT) inhibitors^8^. Most studies utilizing PHH cultures for HBV infection require analysis after up to 15 days, at which time the majority of PHH have already dedifferentiated. Even though the initial infection might have been in PHH, the changing phenotype of cells during culture makes interpretation of results difficult. In vivo however, HBV can be transmitted by low pathogen numbers and in the absence of immunomodulation^12, 13^. This raises the question as to whether a more natural host cell environment including a physiological liver architecture resembling liver sinusoids would impact the susceptibility to infection of PHH with HBV.

Finally, HBV infection has been shown to go largely undetected by the hosts’ immune system, explaining the high viral titers and absence of immune-mediated liver injury during the acute phase of infection. Early experiments in chimpanzees have shown that HBV does not induce a potent innate immune response^13^, and recent reports indicate that HBV might evade detection by interfering with several innate immune-sensing pathways^14^. Most hepatic cell lines and primary cells, however, alter the expression levels of innate immune sensors, which might alter the ability of HBV proteins to interfere with host signal transduction. Indeed, a recent report describes dependency of HBV replication on the absence of a type I interferon (IFN) response^8^, which had not been observed in vivo^13^.

Here we describe a novel 3D microfluidic PHH culture model for HBV, based on a previously developed platform^15–17^. We demonstrate that this culture system recapitulates the hepatic sinusoid microarchitecture including functional bile canaliculi and complete cell polarization. In addition to maintaining stable high levels of albumin secretion, Cyp450 activity as shown by the expression, and activity of phase I, II, and III enzymes for at least 40 days, PHH become susceptible to cell culture- and patient-derived HBV isolates at low MOI and show hallmarks of innate immune activation observed in HBV-infected patients. Comparing this platform to other advanced primary hepatocyte culture models (e.g., hepatic spheroids^18^ or self-assembling co-cultures (SACC PHH)^19^), we show that the 3D PHH cultures enable infection studies at 10,000-fold lower MOI than other advanced culture models. Furthermore, utilizing co-cultures of PHH and primary Kupffer cells (KC) we provide direct evidence that KC fail to recognize HBV infection and do not participate in an early innate immune response. However, upon exogenous stimulation, KC rapidly induce interleukin (IL)-6 and tumor necrosis factor (TNF)-α, which may contribute to the observed suppression of HBV replication.

This preclinical platform opens the door for the identification of biomarkers, dissection of host genetics, and its impact on HBV infection as well as modeling drug treatment responses. The versatility of this novel model system is furthermore underlined by the ability to co-culture innate immune cells including KC, which phenocopy host responses observed in HBV-infected patients.

## Results

### Hepatocytes form hepatic microtissues in 3D culture

**Fig. 1:**
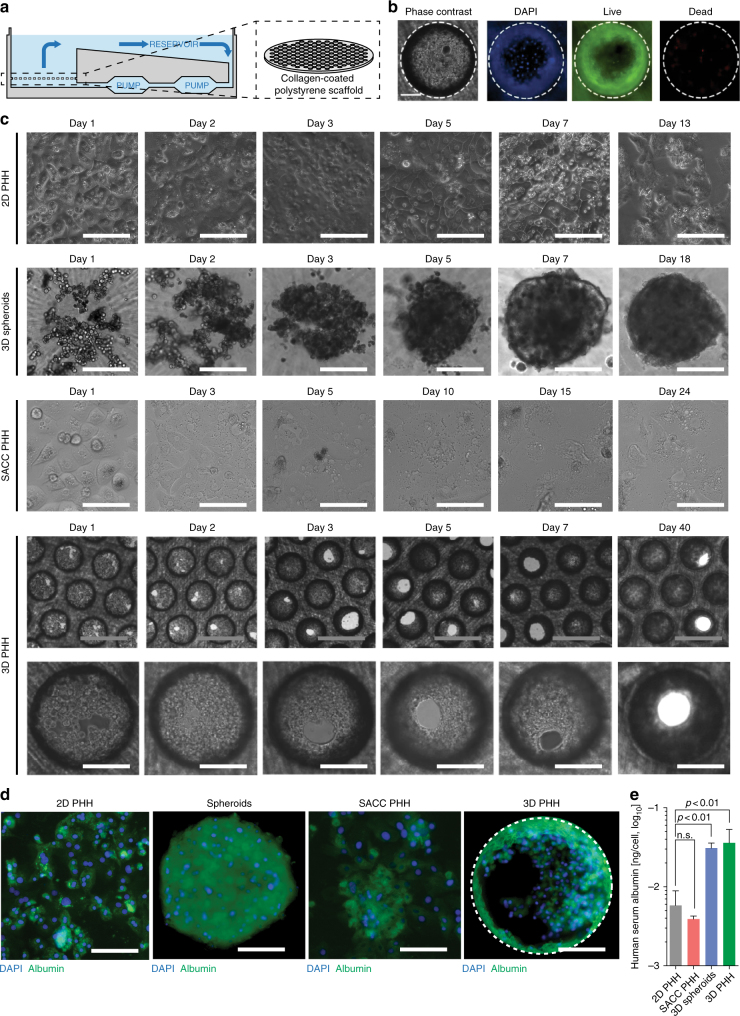
3D PHH cultures form physiological hepatic microtissues for extended periods of time. **a** Schematic of the perfused bioreactor, in which media is recirculated via a pneumatically driven micro-pump, and the collagen-coated scaffold for cell adherence. Each plate consists of 12 individual bioreactors, in which media flow can be regulated. **b** Cell viability of PHH following seeding in 3D scaffolds. Viable cells are stained green with calcein AM while dead cells are stained red with ethidium homodimer-1 3 days post-seeding. **c** Kinetic of hepatic microtissue formation and comparison to 3D spheroid, static 2D PHH, and SACC PHH hepatocyte morphologies. **d** Immunofluorescence microscopy of albumin (green) and DAPI (blue) in 2D PHH, 3D spheroid, SACC PHH, and 3D PHH cultures after 14 days of culture. **e** Secretion of albumin from 2D PHH, 3D spheroid, SACC PHH, and 3D PHH cultures after 14 days of culture as determined by ELISA. All data shown are mean ± SD of three to six independent experiments. *p*-values calculated by two-way ANOVA with Bonferroni post-test. Scale bars: white (200 μm), gray (500 μm)

To model the organization of PHH on liver sinusoids, we used microfluidic recirculation through collagen-coated polystyrene scaffolds seeded with PHH (Fig. 1a). The recirculation of culture media at a speed of 1 μL/s provides nutrients and oxygen to the cultures at stable levels, which is one of the main obstacles of conventional two-dimensional (2D) PHH cultures^15, 16, 20^. In 3D PHH and 3D spheroid cultures, PHH retain viability and exhibit stable morphology and phenotype for at least 40 days (Fig. 1b, c) in contrast to conventional static 2D PHH cultures and SACC PHH (composed by PHH and the mouse fibroblast cell line NIH3T3-J2), which show morphological de-differentiation over 10–13 days. 3D PHH cultures form viable hepatic microtissues (Fig. 1b, c) and produce approximately 10-fold higher levels of the hepatocyte-specific marker albumin, compared to 2D or SACC PHH after 2 weeks of culture (Fig. 1d, e). However, albumin and lactate dehydrogenase (LDH) levels in 3D PHH cultures are comparable to 3D spheroid cultures and remain stable over time (Supplementary Figs. 1, 2 and Supplementary Movie 1).

**Fig. 2:**
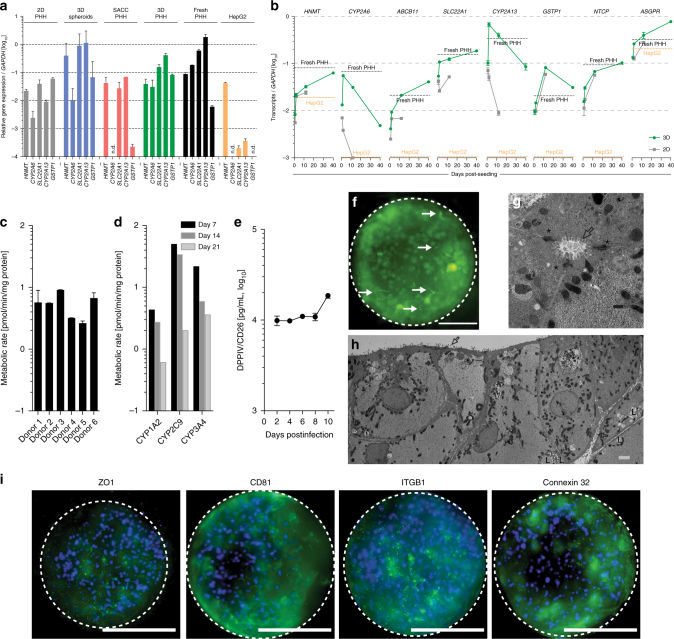
3D PHH cultures are metabolically stable and exhibit functional tissue formation. **a** Comparison of cytochrome P450 gene (HNMT, CYP2A6, SLC22A1, CYP2A13, and GSTP1) mRNA expression between static 2D PHH cultures, 3D spheroid cultures, SACC PHH cultures, and 3D PHH cultures following 14 days of culture as well as freshly thawed PHH and HepG2 cells. **b** Longitudinal comparison of the mRNA expression profile of HNMT, CYP2A6, ABCB11, SLC22A17, CYP2A13, and GSTP1 as well as HBV receptors (NTCP, ASGPR) in HepG2 cells and freshly thawed PHH as well as static 2D and 3D PHH cultures. **c** Determination of CYP3A activity in six different hepatocyte donors 7 days following seeding in 3D PHH cultures as determined by CYP3A-Glo assay. **d** CYP450 activity in 3D PHH following 7, 14, or 21 days of culture time as determined by quantification of metabolites of Tacrine (CYP1A2), Diclofenac (CYP2C9), and Midazolam (CYP3A4) by quadrupole linear ion trap mass spectrometry. **e** Stable maintenance of the bile canaliculi marker DPPIV/CD26 in 3D PHH cultures as determined by Luminex. **f** Functionality of bile canaliculi as determined by CDFDA staining. **g**,** h** Ultrastructural analysis of 3D PHH by transmission electron microscopy after 20 days of culture. (Filled arrows: bile canaliculi, open arrows: hepatic microvilli, asterisk: tight junctions, L: lipid droplet). **i** Immunofluorescence microscopy of tight junction and hepatocyte markers (ZO-1, CD81, ITGB1, Connexin 32) in 3D PHH cultures following 26 days of culture. All data shown are mean ± SD of three to six independent experiments. Scale bars: white (200 μm), gray (2 nm), black (500 nm)

To further characterize the metabolic functionality of 3D PHH cultures, we compared the mRNA levels of key Cyp450 including phase I, II, and III enzymes (HNMT, CYP2A6, ABCB11, SLC22A1, CYP2A13, GSTP1) between PHH grown in 2D, 3D spheroid, SACC PHH, and 3D PHH cultures (Fig. 2a). Overall, cultures grown in 2D exhibited lower or absent levels of Cyp450 genes following 2 weeks of culture (Fig. 2b). Only 3D spheroids and 3D PHH cultures demonstrate Cyp450 levels comparable to freshly thawed PHH. Compared to 2D PHH, 3D PHH cultures are also longitudinally stable in their Cyp450 and HBV entry factor gene expression (NTCP, ASGPR) (Fig. 2b). To test the metabolic activity of 3D PHH, the biological activity of CYP3A in six different hepatocyte donors was evaluated after 7 days of culture and shows uniform CYP3A activity (Fig. 2c). Additionally, the ability of CYP1A2, CYP2C9, and CYP3A4 to metabolize their respective substrates was evaluated 7, 14, and 21 days post-seeding of PHH and suggests the maintenance of functionally intact PHH (Fig. 2d). Stable secretion of DPPIV/CD26, a marker of bile canaliculi^21^ and staining of cultures with 5-CDF furthermore demonstrates that hepatic bile canaliculi are functional and maintained for extended periods of time (Fig. 2e, f). Ultrastructural analysis of PHH grown for 20 days in 3D PHH cultures revealed structural features resembling liver sinusoidal architecture, including bile canaliculi and tight junction formation (Fig. 2g, h, Supplementary Fig. 3). Immunofluorescence microscopy of the antiproliferative antibody 1 (TAPA-1, CD81), connexin 32 (GJB1), integrin β1 (ITGB1), and zona occludens protein 1 (ZO-1) 26 days post-seeding confirm the presence of tight junctions, which are prime indicators for polarized growth of PHH (Fig. 2i, Supplementary Movies 2–5). Tissue formation and longitudinal maintenance of hepatic phenotype are additionally independent of hepatocyte donor, age, or genetic background.

### 3D hepatocytes support long-term HBV infection

**Fig. 3:**
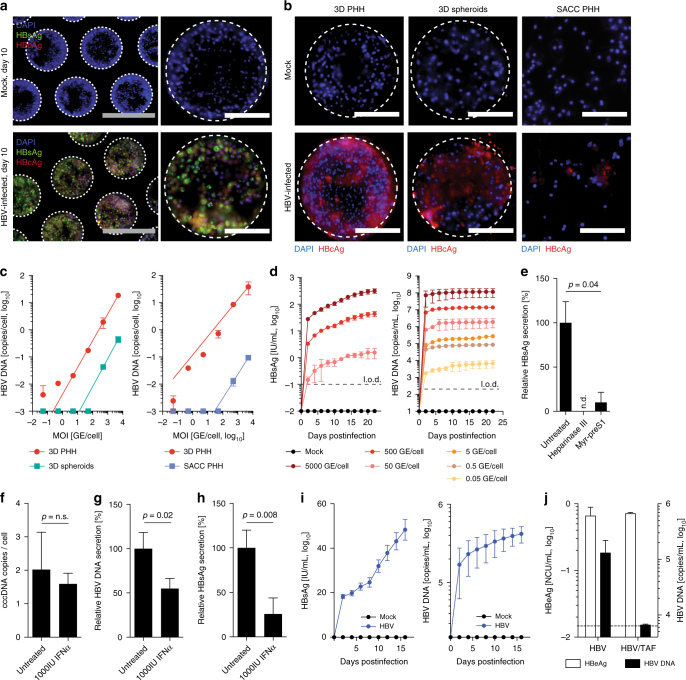
3D PHH cultures are susceptible to long-term HBV infection. **a**, **b** Immunofluorescence detection of **a** HBsAg and HBcAg 10 days following infection of 3D cultures with patient-derived HBV (100 GE/cell) and **b** HBcAg in 3D PHH cultures, 3D spheroid cultures, and SACC PHH cultures 12 days following infection with HepDE19-derived sucrose-purified HBV (500 GE/cell). **c** Susceptibility of 3D PHH cultures compared to 3D spheroid cultures (left) and SACC PHH cultures (right) following infection with HepDE19-derived, sucrose-purified HBV at the indicated MOI via detection of secreted HBV DNA. **d** Longitudinal cumulative HBV DNA and HBsAg secretion following infection of 3D PHH cultures with the indicated MOI of sucrose gradient-purified HepDE19-derived HBV. **e** Blocking of HBV entry in 3D PHH cultures by pretreatment of cells with 5 U/mL heparinase III or myristoylated preS1 peptide (aa2-48, 1 μM) for 1 h prior to infection with 500 GE/cell sucrose-purified HepDE19-derived HBV. HBsAg secretion was determined 6 days postinfection. **f**–**h** Response of HBV-infected 3D PHH cultures to treatment with recombinant IFNα (1000 IU/mL) as determined by quantification of **f** cccDNA copies per cell using quantitative PCR of Hirt-extracted DNA, secretion of **g** HBV DNA, and **h** HBsAg at 21 days postinfection. IFNα treatment was initiated 2 days postinfection and maintained throughout the culture period. **i** Longitudinal cumulative HBsAg and HBV DNA secretion following infection of 3D PHH cultures with 200 GE/cell of patient-derived HBV. **j** HBeAg and HBV DNA secretion of HBV-infected 3D PHH cultures (100 GE/cell) in the presence of 1 μM Tenofovir alafenamide for 10 days. Treatment was initiated 2 days postinfection and was maintained throughout the culture period. All data shown are mean ± SD of three to six independent experiments. *p*-values calculated by two-way ANOVA with Bonferroni post-test. Scale bars: white (200 μm), gray (100 μm)

To evaluate whether 3D PHH can be infected with HBV, cultures were incubated with patient-derived HBV at an MOI of 100 GE/cell followed by detection of HBcAg and HBsAg by immunofluorescence 10 days postinfection, which indicates that the majority of cells are infected (Fig. 3a). HBcAg staining furthermore confirmed that infection was sustained for at least 22 days (Supplementary Movie 6). Direct comparison of the susceptibility of 3D PHH, 3D spheroid, and SACC PHH cultures to different MOIs of sucrose-purified HepDE19-derived HBV furthermore demonstrates that, even though all culture models can be infected with 500 GE/cell (Fig. 3b, c, Supplementary Fig. 4), only 3D PHH cultures still readily become infected, when using as little as 0.05 GE/cell as confirmed by the secretion of HBsAg and HBV DNA (Fig. 3c, d). HBsAg and HBV DNA production was stable for at least 22 days postinfection and, for the first time, demonstrates that 0.05 GE/cell results in stable HBV infection in vitro, even though observable HBsAg secretion at an MOI of 0.05 GE/cell was under the limit of quantification. Despite the ability to detect HBcAg-positive cells in 3D spheroids infected with 50 GE/cell of HBV (Supplementary Movie 7), this infection does not result in sufficient HBV DNA secretion or pgRNA accumulation to be detected by the assays used. In contrast to previously described culture systems for HBV^8, 19^, 3D PHH culture initiation and maintenance of HBV infection does not require the use of PEG or DMSO^8, 19^, does not depend on inhibition of cell-intrinsic innate immune responses^8^, and is independent of host genetic differences between hepatocyte donors (Supplementary Fig. 5a) or the source of patient-derived HBV (Supplementary Fig. 5b).

HBV entry into 3D PHH is still highly dependent on NTCP since preincubation of cultures with a myristoylated preS1 peptide (2–48)^22^ resulted in an inhibition of HBsAg secretion of 90%. Furthermore, depletion of cellular heparan sulfate using heparinase III treatment prior to infection completely abolishes susceptibility to HBV infection, indicating that the attachment of HBV to cellular heparan sulfate is essential to establish infection (Fig. 3e). Additionally, infection of 3D PHH cultures leads to the accumulation and maintenance of sgRNA and pgRNA (Supplementary Fig. 4) as well as cccDNA at approximately 2 copies per cell (Fig. 3f). Treatment of cultures with IFNα demonstrated that cccDNA levels are unaffected, whereas HBV DNA and HBsAg secretion are inhibited by IFNα (Fig. 3g, h), as previously reported^23^. Longitudinal HBsAg and HBV DNA release from 3D PHH cultures infected with patient-derived HBV resulted in a steady increase of both viral markers as well as replicative intermediates (Fig. 3i, Supplementary Fig. 4). When treated with the HBV reverse transcriptase (RT) inhibitor tenofovir alafenamide (TAF), HBV DNA secretion rapidly decreased, whereas antigen expression remained stable, indicating that similar to HBV-infected patients, RT inhibitors are non-curative (Fig. 3j). This demonstrates that 3D PHH cultures are susceptible to HBV at more physiological inoculum doses than 2D PHH, SACC PHH, 3D spheroid cultures, or hepatoma cell lines overexpressing NTCP^24^. Furthermore, we demonstrate that the long-term nature of 3D PHH cultures enables, for the first time, the evaluation of sequential treatments with clinically used drugs (e.g., IFNα, TAF) and novel drug candidates, including Toll-like receptor (TLR) agonists (GS-9620) and epigenetic modulators (C646) (Supplementary Fig. 6). HBeAg is effectively suppressed and HBV DNA decline is more pronounced in sequential treatments using IFNα and TAF in contrast to monotherapy (Supplementary Fig. 6a–d). Using monotherapy with either IFNα or C646 followed by an untreated follow-up time, we furthermore show that both control HBV DNA release (Supplementary Fig. 6g), whereas only C646 additionally reduces the levels of secreted HBeAg and intracellular cccDNA (Supplementary Fig. 6f, h).

This validates 3D PHH cultures as a robust preclinical platform for evaluating novel treatment combinations and subsequent treatment studies.

### HBV suppresses baseline innate immune activation

**Fig. 4:**
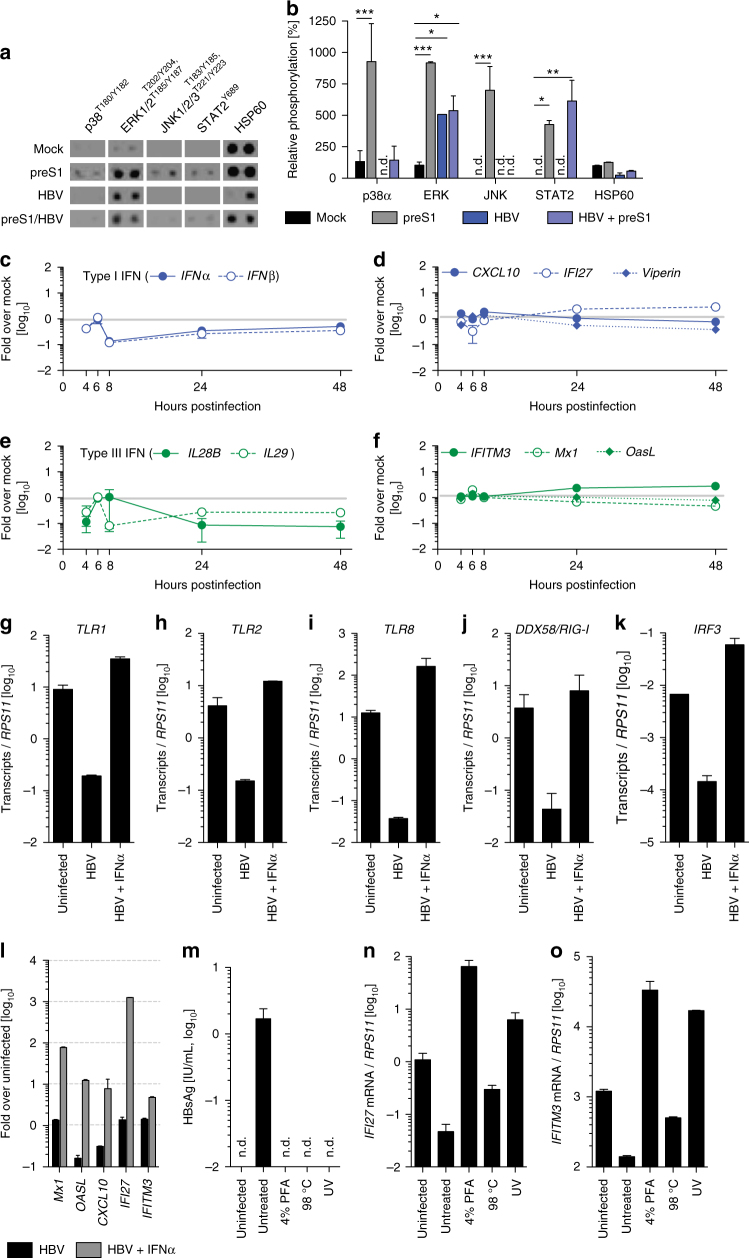
HBV subverts hepatocyte-intrinsic innate immune responses. **a**, **b** Analysis of the induction of p38, ERK1/2, JNK1/2/3, STAT2, and HSP60 phosphorylation following stimulation of uninfected or patient-derived HBV-infected 3D PHH cultures (100 GE/cell) with 1 μM myristoylated preS1 peptide for 24 h using phospho-kinase arrays. Cells were treated/infected 3 days post-seeding of PHH and phosphorylation was determined 24 h later. **c**–**f** Transcriptional profile of **c** type I and **e** III IFN and **d**,** f** ISG within the first 48 h after infection. **g**–**k** mRNA expression of **g** TLR1, **h** TLR2, **i** TLR8, **j** DDX58/RIG-I, and **k** IRF3 in 3D PHH cultures, either left uninfected, infected with 100 GE/cell patient-derived HBV, or HBV-infected cultures treated with 1000 IU/mL IFNα at 10 days postinfection. **l** Expression of ISG in 3D PHH cultures infected with patient-derived HBV (100 GE/cell), either untreated or treated with 1000 IU/mL IFNα at 10 days postinfection. **m**–**o** Inactivation of patient-derived HBV by 4% paraformaldehyde for 1 h, heat treatment at 98 degrees for 2 min, or exposure to UV irradiation for 30 min and resulting innate immune activation as measured by **m** HBsAg secretion 10 days after culture initiation and qPCR of **n** IFI27 and **o** IFITM3. All data shown are mean ± SD of three to six independent experiments (*p < 0.05, **p < 0.01, ***p < 0.001; two-way ANOVA with Bonferroni post-test)

Since previous data indicate that HBV infection in vivo fails to elicit a potent innate immune response^13^, we aimed at dissecting the early host response to HBV infection in 3D PHH cultures. To assess whether 3D PHH cultures maintain the expression levels of key pattern-recognition receptors (PRRs) and signaling mediators, we compared the longitudinal mRNA expression levels of TLRs and cytoplasmic nucleic acid sensors as well as signaling mediators in different hepatocyte donors in 2D and 3D PHH cultures and their levels remained stable for at least 10 days when compared to freshly thawed PHH (Supplementary Fig. 7). Next, we performed phospho-kinase arrays, evaluating the activation of key innate immune pathways by HBV antigen and their suppression by HBV infection (Fig. 4a, b). In particular, we utilized a myristoylated preS1 peptide^25^ to dissect the contribution of HBV binding to its cognate receptor and any other potential PRRs and compared this to productive HBV infection. For this purpose, PHH grown in 3D cultures were either left untreated, treated with the indicated concentration of preS1, infected with 100 GE/cell HBV, or with a combination of HBV infection and preS1 for 24 h. PreS1 peptides result in a pronounced activation of p38, c-Jun N-terminal kinase 1 (JNK1)/2/3, extracellular signal–regulated kinase 1 (ERK1)/2, and STAT2 by phosphorylation 24 h post addition to 3D PHH cultures, whereas HBV infection of 3D PHH cultures only induces detectable levels of ERK1/2 but not p38, JNK1/2/3, or STAT2, indicating that HBV infection interferes with their activation. Strikingly, HBV infection furthermore overrides the potential of preS1 peptides to induce the aforementioned innate immune signaling mediators, since co-administration of HBV-infected 3D PHH cultures with preS1 peptides fails to induce p38, JNK1/2/3, or STAT2. This HBV-mediated interference of innate immune signal transduction furthermore translates to a lack of transcriptional activation of IFN in HBV-infected 3D PHH cultures over time (Fig. 4c, e). Upon infection with HBV, both, type I and III IFN transcripts were suppressed to 10% of the levels observed in uninfected cultures, suggesting an active role for HBV in suppressing innate immune activation. This lack of IFN induction translates to a lack of interferon-stimulated gene (ISG) expression at baseline, including CXCL10, IFI27, Viperin, IFITM3, Mx1, and OasL following HBV infection (Fig. 4d, f). Cells, however, retain their responsiveness to exogenous IFNα, as indicated by the induction of ISGs (Fig. 4l), suggesting that HBV exerts its immunomodulatory function prior to the induction of IFN. However, HBV infection suppresses the expression of TLRs 1, 2, and 8; RIG-I; and IRF3 (Fig. 4g–k) 10 days postinfection, suggesting that, in addition to the direct impact on signal transduction, HBV impairs innate immune responses at the transcriptional level. This suppression of innate immune sensors and mediators was completely reversed by treatment with IFNα. To further investigate as to whether the observed suppression of innate immune activation is directly exerted by HBV, we compared induction of cytokine responses following infection with HBV or with ultraviolet (UV)-, heat-, or paraformaldehyde-inactivated HBV. UV inactivation of HBV abolishes the block in innate immune activation, as evident by the increased levels of IFI27 and IFITM3 mRNA in the absence of HBV replication 10 days postinfection, supporting that direct immune evasion of HBV occurs early during infection (Fig. 4m–o). Even though all inactivation conditions used resulted in complete loss of infectivity, the ability of a viral antigen to exert immune activation in 3D PHH cultures depends on conformational integrity since heat-induced antigen denaturation does not induce potent immune activation.

### 3D PHH phenocopy host responses seen in HBV-infected patients

**Fig. 5:**
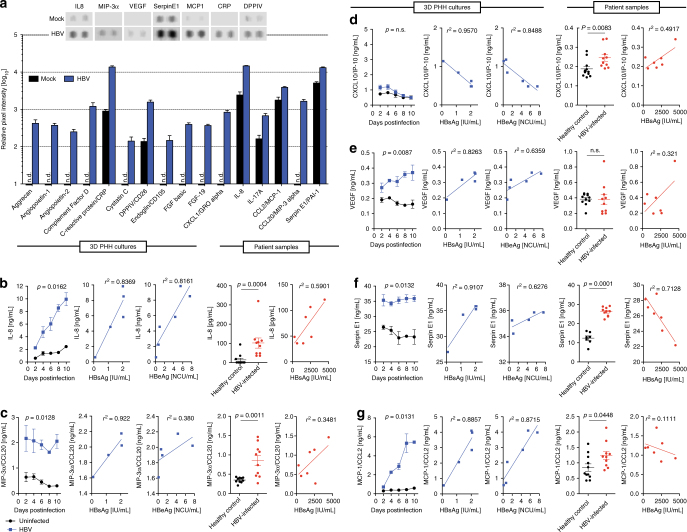
HBV-infected 3D PHH cultures predict human host responses. **a** Cytokine XL antibody array of uninfected 3D PHH cultures and cultures infected with 100 GE/cell patient-derived HBV for 10 days. **b**–**g** Comparison of the longitudinal protein expression values of **b** IL-8, **c** MIP-3α, **d** CXCL10, **e** VEGF, **f** Serpin E1, and **g** MCP-1 between HBV-infected patients, healthy controls, and 3D PHH cultures infected with patient-derived HBV (100 GE/cell) as well as correlation of each cytokine with HBsAg (HBV-infected patients and infected 3D PHH cultures) and HBeAg (infected 3D PHH cultures) levels. Data shown are mean ± SD of three independent experiments. *p*-values calculated by two-way ANOVA with Bonferroni post-test

To determine whether the host response to HBV infection could be translated to that observed in HBV-infected patients, we measured global changes in chemokine and cytokine responses in 3D PHH cultures following HBV infection, utilizing Cytokine XL antibody arrays, which simultaneously determine changes in over 100 cytokines and chemokines. As evident, HBV infection does not induce a pronounced proinflammatory response as evidenced by the lack of induction of IL-1b, IL-6, IL-12, and the majority of other cytokines and chemokines (Fig. 5a). To compare host responses in HBV-infected 3D PHH cultures to serum samples from HBV-infected patients (Supplementary Table 2), we performed Luminex protein analysis. Even though 3D cultures solely contain PHH and no other cell types, levels of IL-8, macrophage-inflammatory protein (MIP)-3α, SerpinE1, and monocyte chemotactic protein-1 (MCP-1) showed significant elevation on the protein level in HBV-infected 3D PHH cultures as well as in the sera of HBV-infected patients (Fig. 5b, c, f, g). Vascular endothelial growth factor (VEGF), which was highly induced in 3D PHH cultures, was not elevated in the sera of HBV-infected patients, which may be due to the short radius of action of VEGF, restricting it to the liver (Fig. 5e). C-X-C motif chemokine ligand 10 (CXCL10), which is produced predominantly by plasmacytoid dendritic cells^26^, demonstrated an inverse correlation with HBsAg and HBeAg and levels detected are far lower than those observed in HBV-infected patients, further supporting that in PHH, ISG induction is inhibited by HBV (Fig. 5d). Notably, both in 3D PHH cultures and HBV-infected patient sera, the levels of IL-8 and MIP-3α correlated with HBsAg levels, suggesting a direct effect of HBV infection. In 3D PHH cultures, the direct correlation also exists with secreted HBeAg levels. These factors were also shown to be elevated in the serum of patients infected with HBV in clinical studies^27–30^. Taken together, these results indicate that 3D PHH accurately recapitulate host responses observed in patient samples and earlier studies using chimpanzees^13^, validating 3D PHH cultures for the identification and dissection of host/pathogen interactions and biomarkers.

### Co-cultures with KC do not impact HBV infection

**Fig. 6:**
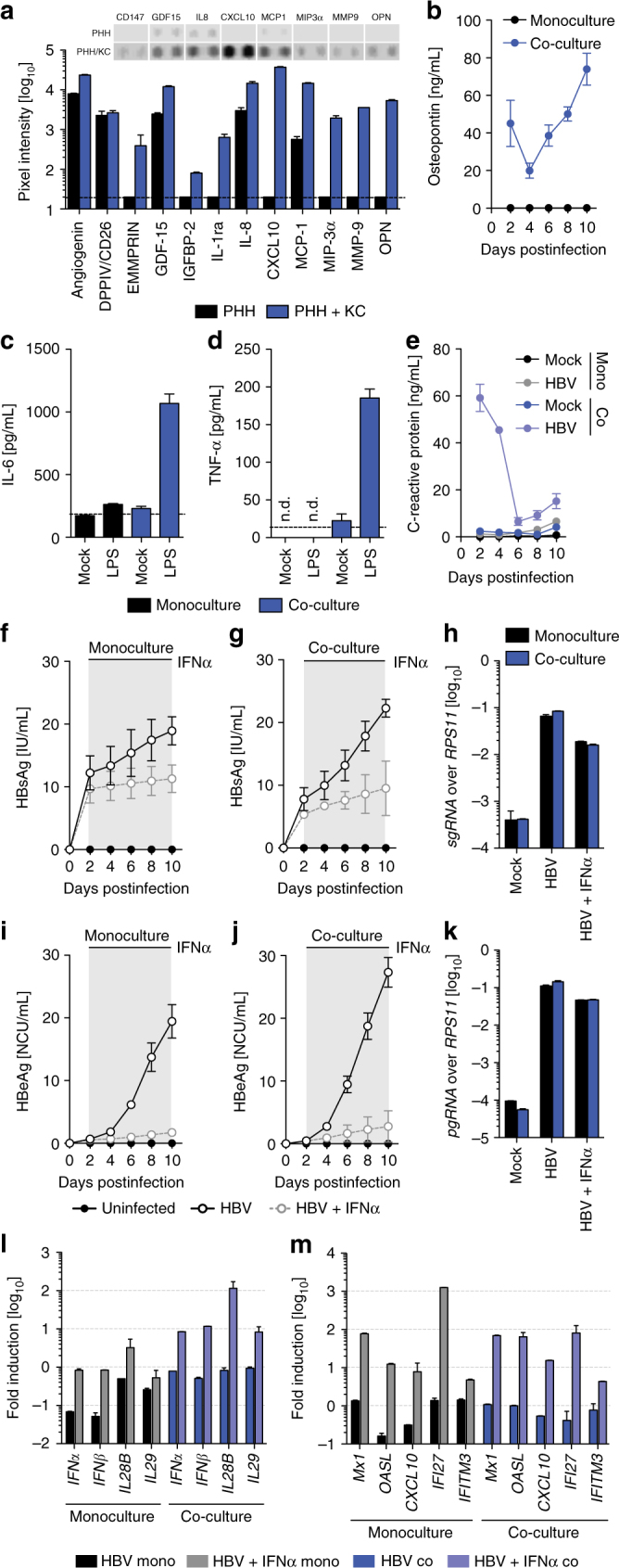
Kupffer cells do not alter HBV infection kinetics in 3D PHH cultures. **a** Cytokine XL antibody array of 3D PHH and 3D PHH/KC co-cultures (ratio 10:1) 13 days post-seeding. **b** Longitudinal osteopontin protein levels in 3D PHH and 3D PHH/KC co-cultures, determined by Luminex. **c**,** d** Response of 3D PHH and 3D PHH/KC co-cultures to 1 μg/mL LPS added 11 days post-seeding as determined by measurement of **c** IL-6 and **d** TNF-α 24 h post stimulation. **e** Acute phase response elicited by KC following infection with patient-derived HBV (100 GE/cell), determined by measurement of C-reactive protein using Luminex. **f**–**k** Comparative infection of **f**,** h**,** i**,** k** PHH and **g**,** h**,** j**,** k** PHH/KC co-cultures with patient-derived HBV (100 GE/cell) as determined by cumulative secretion of **f**,** g** HBsAg and **i**,** j** HBeAg and intracellular accumulation of **h** subgenomic HBV RNA and **k** pregenomic RNA. **l**,** m** Transcription of **l** type I and III interferon and **m** ISG following 10 days of infection of PHH or PHH/KC co-cultures with patient-derived HBV (100 GE/cell) in the absence or presence of 1000 IU/mL exogenous IFNα. Data shown are mean ± SD of four independent experiments

Even though 3D PHH cultures are able to recapitulate many of the host responses described in humans, many interactions of HBV and its surrounding host involves other liver-resident cells. It has previously been shown that IL-6, which is predominantly produced by macrophages, plays an important role in suppressing HBV transcriptional activity^31, 32^. However, no expression of IL-6 or TNF-α was detectable even though HBV-infected patients exhibit elevated levels of both cytokines (Fig. 6). To address the impact of non-parenchymal cell populations within the liver on HBV infection, we performed co-cultures of 3D PHH and primary KC cells. We used PHH monocultures and co-cultures with a PHH:KC cell ratio of 10:1 and analyzed the cytokine and chemokine secretome 13 days post-seeding using Cytokine XL antibody arrays (Fig. 6a) and longitudinal cell viability via albumin secretion and LDH release (Supplementary Figs. 2, 8). In contrast to 3D PHH, 3D PHH/KC co-cultures stably release EMMPRIN (CD147), IGFBP-2, IL-1ra, CXCL10/IP-10, MIP-3α, matrix metalloproteinase-9 and Osteopontin (OPN) (Fig. 6a). Longitudinal analysis of OPN expression levels in monocultures and co-cultures confirmed that KC remain viable for at least 13 days post-seeding (Fig. 6b). To evaluate the functionality of KC, we stimulated 3D PHH and 3D PHH/KC co-cultures with 1 μg/mL lipopolysaccharide (LPS) 11 days post-seeding and analyzed the protein levels of IL-6 and TNF-α at day 12. As expected, only co-cultures secreted high levels of IL-6 and TNF-α, indicating that KC remain functional throughout the culture period (Fig. 6c, d). Infection of 3D PHH and 3D co-cultures with 100 GE/cell HBV resulted in a pronounced acute phase response, as evident by high levels of C-reactive protein only in PHH/KC co-cultures (Fig. 6e), which has been shown to be produced by macrophages^33^.

Comparing virological characteristics of HBV infection revealed that the secreted levels of HBsAg and HBeAg, and levels of pgRNA and sgRNA, are identical in monocultures and co-cultures (Fig. 6f–k). Treatment of both monocultures and co-cultures with IFNα resulted in the reduction of viral replication to similar extents as indicated by the reduction of secreted HBsAg, HBeAg, pgRNA, and sgRNA (Fig. 6f–k). In contrast to PHH monocultures, the presence of KC abolished the HBV-induced suppression of IFN production (Fig. 6l). This elevation in type I and III IFN was, however, likely to originate from the KC, since HBV infection in PHH/KC co-cultures still failed to elicit a potent ISG response (Fig. 6m), suggesting that, even though they remain functional in culture, they do not actively interfere with HBV or its ability to subvert innate immune responses.

### Exogenous stimulation of KC suppresses HBV replication

**Fig. 7:**
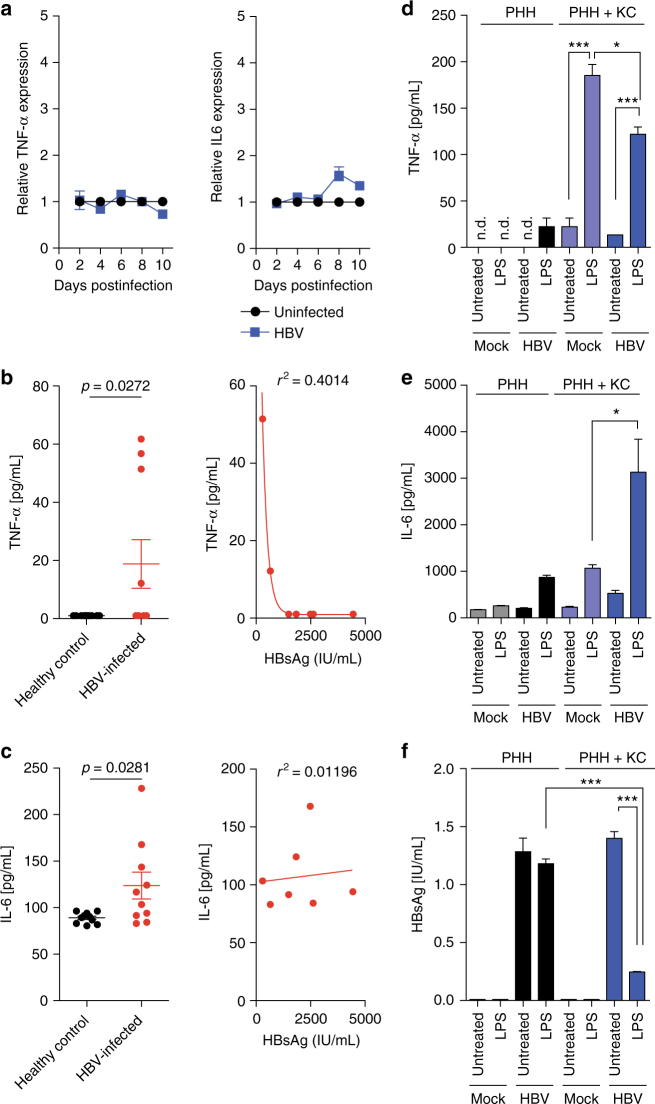
Exogenous signals are required for Kupffer cell responses to HBV infection. **a** Longitudinal secretion of TNF-α and IL-6 in 3D PHH cultures infected with patient-derived HBV (100 GE/cell). **b**,** c** Serum levels of **b** TNF-α and **c** IL-6 as well as correlation with serum HBsAg levels of HBV-infected patients and healthy controls. **d**–**f** Secretion of **d** TNF-α and **e** IL-6 as well as **f** HBsAg in 3D PHH cultures and 3D PHH/KC co-cultures infected with patient-derived HBV (100 GE/cell). Cultures were treated with 1 μg/mL LPS 8 days postinfection, TNF-α and IL-6 levels were quantified by Luminex 24 h post LPS stimulation, and HBsAg secretion was determined 48 h post LPS stimulation. Data shown are mean ± SD of three independent experiments (*p < 0.05, ***p < 0.001; two-way ANOVA with Bonferroni post-test)

Since KCs do neither affect HBV replication nor mount proinflammatory immune responses, we evaluated as to whether KCs still respond to exogenous stimuli. 3D PHH cultures themselves do not produce any IL-6 and TNF-α in response to infection with HBV since these cytokines are KC specific (Fig. 7a). However, HBV-infected patients exhibit elevated serum levels of IL-6 and TNF-α, suggesting proinflammatory immune responses (Fig. 7b, c). Analysis of the secretion of cytokines and chemokines revealed that HBV-infected co-cultures, similar to 3D PHH monocultures, do not secrete IL-6 and TNF-α in response to HBV infection (Fig. 7d, e). However, when exogenous stimulation is applied via LPS even 8 days after infection with HBV, KC rapidly secrete IL-6 and TNF-α (Fig. 7d, e). Interestingly, LPS-induced IL-6 responses were significantly stronger in HBV-infected compared to uninfected cultures, suggesting that, even though HBV effectively blocks most innate immune activation in PHH, it is not able to subvert exogenous activation of non-parenchymal cells. This LPS-induced innate immune activation is furthermore able to suppress HBV replication, as determined by reduced HBsAg secretion (Fig. 7f) in the absence of LPS-induced cytotoxicity or IL-6-induced proliferation, as determined by albumin secretion (Supplementary Fig. 8), suggesting that exogenous antigen may be involved in eliciting immune responses to HBV infection. This demonstrates that 3D PHH/KC co-cultures can be utilized to dissect the contribution of individual liver-resident cell types to antiviral responses against HBV.

## Discussion

The study of HBV infection has been complicated in the past by the unavailability of model systems allowing the study of disease pathogenesis and limiting research to using patient samples or animal models. Even though many advanced culture platforms for PHH have been developed in recent years^9, 10, 34–39^, no information is yet available on their susceptibility to HBV infection. The discovery of NTCP as entry factor into human hepatocytes has paved the way to facilitating molecular studies of the complete HBV life cycle, but the downregulation of innate immune factors in cell lines restricts the study of host/pathogen interactions and disease pathogenesis to PHH cultures. Most 2D PHH cultures, even though permitting short-term studies, are difficult to maintain and rapidly de-differentiate over the course of several days^11^. Here we describe a novel 3D PHH culture system facilitating the study of HBV under physiological conditions and compare it to conventional 2D PHH, 3D hepatic spheroid, and SACC PHH cultures. The constant recirculation of nutrients and oxygenated medium leads to the formation of hepatocyte microtissues, which are metabolically and functionally stable for at least 40 days post-seeding, allowing the study of long-term effects of HBV on PHH and the analysis of sequential drug treatments in the context of HBV infection. Microtissues are characterized by the formation of a liver-like microarchitecture including tight junctions and bile canaliculi, which localizes host factors at correct cellular surfaces. This is especially important for HBV, where receptors are distinctly expressed on basolateral hepatocyte surfaces^24^. The 2D nature of PHH cultures results in only limited cell polarization with random cell surface distribution of entry factors, potentially explaining the poor susceptibility of 2D cultures to HBV infection^11^. The nearly equal amounts of NTCP mRNA in 2D and 3D PHH cultures would suggest that infection should be possible to equal extent, given that mRNA expression correlates with protein expression. This raises the question whether NTCP and ASGPR represent the sole receptors for HBV on PHH.

It has been previously shown that most 2D systems for HBV, including HepG2/hNTCP, HepaRG, and 2D PHH cultures, require high inoculation titers to establish infection, limiting their use to study patient-derived viral isolates. Even using more advanced model systems incorporating the micropatterning of PHH on collagen and the use of murine feeder cells only marginally raises the hepatocyte susceptibility to HBV infection, while requiring active suppression of innate immune signaling pathways in order to detect significant levels of HBV antigen. We demonstrate that 3D spheroids susceptibility to HBV infection is lost below 500 GE/cell of inoculum, even though some HBcAg-positive cells are still detectable at MOI 50. Most of these HBV infection systems furthermore rely on the addition of large quantities of DMSO, PEG^11^, or immunomodulatory agents^8^ to detect infection. This is in stark contrast to HBV infection in vivo, where minimal HBV inocula are sufficient to establish infection^13^. We demonstrate that hepatocyte polarization is critical for their susceptibility to HBV infection since as little as 0.05 GE/cell can initiate stable infection for at least 22 days, whereas the same HBV inoculum does not result in any detectable HBV infection using 2D cultures, 3D spheroids, or SACC PHH cultures. Notably, all tested PHH donors and HBV patient samples were able to initiate and maintain infection in 3D PHH cultures, independent of whether they were HBeAg-positive or -negative. In contrast to other HBV culture systems, HBV DNA, HBsAg, and HBeAg secretion are rapid and stable over time, resulting in the accumulation of cccDNA. Every hepatocyte in infected cultures harbors approximately two cccDNA molecules, which is similar to the number previously reported in human liver biopsies. Even though infected cultures respond to IFNα treatment with reduced HBV DNA and HBsAg secretion, cccDNA levels remain largely unaffected.

Since, in contrast to traditional 2D PHH cultures, all tested PHH donors are susceptible to infection using this 3D PHH culture system, this opens the door for comparative studies on host genetic effects on HBV. Especially in light of a thus far poorly understood contribution of IL-28b genotypes to HBV infection outcome, the ability to compare PHH donors with differential innate immune responses might reveal important mechanisms of immune control^40^. Most importantly, 3D PHH cultures can be used for infections with patient-derived HBV, which could not be achieved using 2D PHH cultures or HepG2/hNTCP. This offers the unique opportunity for comparative analysis of different HBV genotypes, HBeAg-positive and -negative isolates, drug-resistant strains, and mutants with deletions within the HBV genome. Similar to other HBV culture systems, including HepG2/NTCP cells, HepaRG cells, 2D PHH cultures, 3D spheroids, and SACC PHH cultures, no viral spread to neighboring, uninfected cells, was visible, since low MOI infections failed to spread through the cultures, as evidenced by HBsAg and HBV DNA secretions not reaching levels observed in high MOI infections. This indicates that additional host factors or cell populations may be involved in the spread of HBV, which ultimately results in >80% of the human liver becoming infected with HBV.

The ability of 3D PHH cultures to maintain hepatocyte morphology for extended periods of time additionally enables the evaluation of subsequent treatments in order to inform combination clinical trials. As previously demonstrated in clinical trials^41, 42^, sequential treatment with IFNα and a nucleotide analog resulted in a more profound viral suppression at earlier time points compared to either monotherapy. Furthermore, using recently described epigenetic modulators^43^ we demonstrate that inhibition of p300 using C646 results not only in an early suppression of HBV antigen expression and HBV DNA release but also reduces the levels of cccDNA.

Most hepatoma cell lines as well as PHH in 2D either alter their expression of key innate immune receptors or loose expression rapidly following plating on collagen-coated plates, hindering the evaluation of physiological host responses. As we show, PHH grown in 3D microfluidic cultures do express and maintain stable expression levels of innate immune receptors and downstream effectors, enabling the study of host/pathogen interactions. HBV, however, elicits minimal-to-no innate immune activation in 3D PHH cultures, despite the stable expression of all relevant DNA and RNA sensors usually required for viral sensing. This may be mainly due to effective innate immune inhibition by HBV^44–47^, including the HBV RT^48^ and the X protein^49^, but might also stem from hepatocytes not being professional pathogen sensing and antigen-presenting cells. Since HBV specifically evolved to evade pattern recognition, initiated by HBV components, which would activate innate immune responses, the observed dependence on secondary stimuli through PRRs detecting bacterial products could be attributed to a lack of evolutionary pressure to evade bacteria-initiated innate immunity. HBV infection in 3D PHH cultures results in active suppression of type I and III IFN production and subsequent reduction of ISG expression. Interestingly, this extends to CXCL10, which has previously been shown to be induced by HBV in 2D PHH cultures^50^. This may indicate that HBV actively subverts CXCL10 production and the lower infection efficiency of 2D PHH cultures allows for its production by uninfected bystander cells. However, HBV infection does not interfere with IFN signaling since IFNα readily induces high levels of ISGs, irrespective of whether they are infected with HBV or not. The active suppression of innate immune responses by HBV is furthermore supported by the loss of innate immune evasion following inactivation of HBV. Nevertheless, HBV infection of 3D PHH results in alterations in the cytokine and chemokine fingerprint of infected cultures, which correlates with those observed in HBV-infected patients. In addition to allowing the identification of biomarkers correlating with HBV infection in PHH, 3D cultures are amenable to co-cultures of PHH with other non-parenchymal cells, in particular human KC. For the first time, this enables the dissection of the cellular origin of biomarkers associated with HBV infection. Remarkably, KC fail to respond to HBV antigen by themselves, resulting in uncontrolled replication of HBV, even though they elicit a potent acute phase response. KC secrete large quantities of IL-6 and TNF-α only in response to a secondary stimulus like LPS, which in turn actively suppress HBV replication. This suggests that HBV efficiently silences not only hepatocyte-intrinsic innate immune activation processes but also effectively evades the immunosurveillance function of liver-resident macrophages. Recently, several immunomodulatory functions of HBV proteins have been identified^44^. Among them, several are directly targeting innate immune activation pathways, and even though HBV-infected patients exhibit elevated levels of both IL-6 and TNF-α, further investigation of the impact of HBV or HBV proteins directly on KC is needed.

Taken together, we provide a novel culture model for HBV that allows inoculation of PHH with low-titer patient-derived HBV. In addition to facilitating the study of host and viral genetics, this platform also enables the dissection of complex host/pathogen interactions and validation of biomarkers of HBV infection, treatment responses, or potential curative therapeutic interventions using PHH/KC co-cultures, which cannot be studied using other in vitro or even in vivo xenograft systems. This opens the door to further expanding this culture platform to other non-parenchymal cells to form a “liver on a chip”, applicable not only to the study of HBV but also to other hepatotropic pathogens, liver biology, and drug development.

## Methods

### Cells

HepDE19 cells were kindly provided by Haitao Guo (Indiana University, IN, USA) and were maintained as described^51^. Cryopreserved PHH were provided by QPS Laboratories (Newark, NJ, USA) and Life Technologies (Carlsbad, CA, USA) and KC were from Life Technologies (Supplementary Table 1). HepG2 cells (ATCC, Middlesex, UK) were cultured in Dulbecco’s modification of Eagle’s medium (DMEM) (Gibco, Thermofisher, Paisley, UK) containing 10% fetal bovine serum (FBS; Gibco, Thermofisher), 100 IU/mL Penicillin, and 100 μg/mL Streptomycin (Merck, Millipore, Hertfordshire, UK) in a 5% CO_2_ incubator at 37 °C. PHH and KC were removed from liquid nitrogen and thawed according to the manufacturer’s instruction. Briefly, PHH were resuspended in prewarmed thawing medium (QPS Laboratories). PHH were centrifuged at 100 × *g* for 10 min and resuspended in Williams E medium (WEM) supplemented with Thawing/Plating Supplement Pack (Invitrogen, Paisley, UK) that consisted of 5% FBS, 1 μM Dexamethasone, 100 IU/mL Penicillin, 100 μg/mL Streptomycin, 4 μg/mL Human Recombinant Insulin, 2 mM GlutaMAX^TM^, and 15 mM HEPES pH 7.4. KC were resuspended in Advanced DMEM medium supplemented with Thawing/Plating Supplement Pack (Invitrogen) without Dexamethasone. KC were centrifuged at 500 × *g* for 5 min and resuspended in complete Advanced DMEM medium. Cell viability was determined using trypan blue (Sigma-Aldrich, Dorset, UK) and was >90% in all cases.

### Reagents

IFNα was purchased from Invitrogen; TAF, C646, and GS-9620 were purchased from Selleck Chemicals (Houston, TX, USA). Antibodies against HBsAg (ab20930, 1:100) were from Abcam (Cambridge, UK), and HBcAg (B0586, 1:200) were from Dako (Cambridgeshire, UK). Antibodies against CD81 (TAPA-1, JS-81) were from BD Biosciences (555675, 1:200) and antibodies recognizing connexin 32 (ab66613), ITGB1 (ab30394), and ZO-1 (ab59720) were from Abcam (all 1:100). The antibody recognizing human albumin was from Fitzgerald (10-1950, 1:200, Acton, MA, USA). Alexa Fluor488®-, Alexa 594®- and Alexa700®-conjugated secondary antibodies (A-11034, R37117, A-21038) were from Life Technologies (all 1:2000). LPS was obtained from Invivogen (San Diego, CA). The myristoylated preS1 peptide (aa2–48) was obtained from Thermo Fisher Scientific. Heparinase III was obtained from Sigma Aldrich.

### Microfluidic 3D culture system

Cells were seeded into isolated bioreactors within the LiverChip platform (CNBio Innovations, Welwyn Garden City, Hertfordshire, UK) with media flow in the downward direction on top of collagen-coated scaffolds for 8 h at a flow rate of 1.0 µL/s. Following cell attachment within the scaffold, the flow was changed to the upward direction and maintained at 1.0 µL/s for the remainder of the culture. Hepatocyte monocultures were seeded at a density of 0.6 × 10^6^ viable cells in 1.6 mL of medium per well. The cells were maintained in WEM supplemented with Thawing/Plating Supplement Pack (Invitrogen) for the first 24 h of culture and in WEM supplemented with Cell Maintenance Supplement Pack (Invitrogen) thereafter for the duration of the culture. The media was replaced every 48 h. PHH/KC co-cultures were seeded simultaneously, at a ratio of 10:1. Co-cultures were seeded in Advance DMEM supplemented with Thawing/Plating Supplement Pack without dexamethasone (Invitrogen) for the first 72 h of culture and then in WEM supplemented with Cell Maintenance Supplement Pack without dexamethasone and with 100 nM hydrocortisone (Invitrogen). The media was replaced every 48 h.

### 2D static hepatocyte cultures

PHH were seeded in collagen-coated 12-well plates at a density of 600,000 cells/well in WEM supplemented with Thawing/Plating Supplement Pack (Invitrogen). After 24 h, medium was replaced with WEM supplemented with Cell Maintenance Supplement Pack (Invitrogen) and total medium was replaced every 48 h.

### 3D hepatocyte spheroid cultures

3D spheroid cultures were performed as previously described^18^. Cells were seeded into 96-well ultra-low attachment plates (Corning, Amsterdam, The Netherlands) at a density of 1500 cells/well and were maintained in WEM supplemented with Thawing/Plating Supplement Pack (Invitrogen) for 5 days prior to HBV infection until the spheroid formation is readily detectable by microscopy. Medium was subsequently replaced with WEM supplemented with Cell Maintenance Supplement Pack (Invitrogen) and 50% of the media volume was replaced every 48 h.

### Self-assembling co-cultures of PHH

PHH were seeded at a density of 30,000 cells/well in collagen-coated 96-well plates (Corning) in WEM supplemented with Thawing/Plating Supplement Pack (Invitrogen). After 24 h, 15,000 NIH3T3/J2 cells in WEM supplemented with Cell Maintenance Supplement Pack (Invitrogen) were added and the medium was changed every 48 h for 10 days before infection with HBV. DMSO 0.5% was added to the cultures 24 h before HBV infection as previously described^19^.

### HBV production and purification

Infectious recombinant HBV was produced from the tetracycline-inducible HepDE19 cells. Cells were seeded at 80% confluence in Millicell HY 5-layer cell culture flasks (Millipore), and from 8 days post withdrawal of tetracycline to induce the production of HBV, virus-containing supernatants were harvested every 48 h for 12 days. HBV was precipitated from supernatants using a total concentration of 6% PEG for 16 h at 4 °C and purified by sucrose density gradient purification using an SW28 rotor at 140,000 × *g* for 16 h at 4 °C using a Beckman XPN ultracentrifuge (High Wycombe, UK). The resulting virus was resuspended in phosphate-buffered saline (PBS) containing 10% FBS and stored at −80 °C.

### HBV patient-derived viruses and infections

HBV-positive serum samples of five different donors were used at the indicated MOI. For the 3D infections, serum samples were diluted in 1.6 mL of WEM supplemented with Cell Maintenance Supplement Pack with dexamethasone or hydrocortisone (Invitrogen). HBV infection was performed 3 days after seeding for 24 h, after which cells were washed three times with maintenance medium for 3.5 min using 1 µL/s downward flow. 2D infections were performed using PHH seeded in 24-well collagen-treated plates. Infections were carried out for 24 h, after which cells were washed three times and the medium was replaced with maintenance medium.

### Viral inactivation

HBV inactivation was accomplished by either heat inactivation at 98 °C for 1.5 min, UV inactivation (254 nm, 3 J/cm^2^), or by incubation with a final concentration of 4% *w*/*v* formaldehyde for 1 h at room temperature. Formaldehyde was removed by filtration using Amicon Ultra Centrifugal filter units (Millipore).

### Cell viability analysis

CytoTox 96® Non-Radioactive Cytotoxicity Assay Kits and Celltiter Glo Cell Viability Kits were purchased from Promega (Madison, WI, USA), and the assay was performed according to the manufacturer’s protocol. Absorbance (490 nm) was measured using a BMG Labtech FluoStar Optima Plate Reader. Calcein AM and ethidium homodimer-1 were from Thermo Scientific.

### Human albumin enzyme-linked immunosorbent assay

Human serum albumin enzyme-linked immunosorbent assay (ELISA) was performed as previously described^52^. Briefly, ELISA plates were coated with goat-anti-human-albumin primary antibody (1:400 at 37 °C for 2 h, A80-129A from Bethyl, TX, US) and blocked using 1% bovine serum albumin fraction V (Sigma) in PBS at 37 °C for 1 h. Supernatants from the cultures were diluted and incubated at 37 °C for 1 h. Mouse monoclonal anti-human albumin antibody (ab399, Abcam) was used for primary detection (1:1000 at 37 °C for 2 h) and a horseradish peroxidase (HRP) goat anti-mouse IgG secondary antibody (405306, BioLegend, London, UK) was used for secondary detection (1:2000 at 37 °C for an hour). TMB ELISA substrate was added to measure peroxidase activity. The colorimetric reaction was stopped by adding 1 M H_2_SO_4_. Absorbance (450 nm) was measured using a BMG Labtech FluoStar Optima Plate Reader.

### HBsAg and HBeAg ELISAs

HBsAg and HBeAg levels in cell culture supernatants were quantified using CLIA ELISA Kits (Autobio Diagnostic, Zhengzhou, China), according to the manufacturer’s protocol.

### Cytokine XL and phospho-kinase arrays

To quantify the expression of cytokines and chemokines, a Proteome Profiler™ Human XL Cytokine Array Kit, a membrane-based antibody array for the determination of relative levels of 102 human cytokines and chemokines, was used according to the manufacturer’s protocol (R&D Systems, Inc., USA). Briefly, cell culture supernatants, harvested 10 days post HBV infection at different conditions, were incubated overnight with the Proteome Profiler Human XL Cytokine Array. The membrane was washed to remove unbound material followed by incubation with a cocktail of biotinylated detection antibodies. Streptavidin-HRP and chemiluminescent detection reagents were then applied to produce a signal at each capture spot corresponding to evaluate the amount of protein bound. To quantify the phosphorylation profiles of kinases and their protein substrates, a Proteome Profiler™ Human Phospho-Kinase Array, a membrane-based antibody array for the parallel determination of the relative levels of human protein kinase phosphorylation, was used according to the manufacturer’s protocol (R&D Systems, Inc., USA). Briefly, cell lysates were diluted and incubated overnight with the Human Phospho-Kinase Array. The array was washed to remove unbound proteins followed by incubation with a cocktail of biotinylated detection antibodies. Streptavidin-HRP and chemiluminescent detection reagents were applied to produce a signal at each capture spot corresponding to evaluate the amount of phosphorylated protein bound.

### Luminex analysis

Cytokines identified as differentially regulated using Proteome Profiler™ Human XL Cytokine Array were validated using Luminex bead arrays of IL-8, CXCL10, MCP-1, MIP-3α, Serpin E1, IL-6, TNF-α, Osteopontin, DPPIV, and VEGF according to the manufacturer’s protocol (R&D Systems, Inc., USA). Data were analyzed using a five-parameter logistic curve fit.

### HBV DNA extraction and quantification

DNA from supernatants harvested at different time points was extracted using the DNeasy Blood & Tissue Kit (Qiagen, Manchester, UK) according to the manufacturer’s protocol. Then viral load was evaluated by quantitative PCR (qPCR) as described^53^. The primer sequences used are forward primer: 5ʹ-GTGTCTGCGGCGTTTTATCA-3ʹ and reverse primer: 5ʹ-GACAAACGGGCAACATACCTT-3ʹ from Life Technologies, and probe: 5ʹFAM-CCTCTKCATCCTGCTGCTATGCCTCATC-3ʹTAMRA from Life Technologies. Quantification was normalized to a 1.3×HBV-containing plasmid (pCMV-HBV), kindly provided by Christoph Seeger (Fox Chase Cancer Center, PA, USA).

### HBV cccDNA extraction and quantification

The quantification of cccDNA was performed as described previously^54^. Briefly, total DNA was isolated from scaffolds using the DNeasy Blood & Tissue Kit (Qiagen), subjected to Plasmid-Safe™ ATP-Dependent DNase digest (EpiCentre, Madison, WI, USA), and amplified using qPCR primers specific to the single-stranded HBV genomic region. The primer sequences used are forward primer: 5ʹ-ATCTGCCGGACCGTGTGC-3ʹ and reverse primer: 5ʹ-TTGGAGGCTTGAACAGTAGGA-3ʹ from Life Technologies, and probe: 5ʹFAM-GCACGTCGCATGGAGA-3ʹTAMRA from Life Technologies. Quantification was normalized to a plasmid containing the complete 951–1621 bp region unique to cccDNA of 3.2 kb size, generated through Life Technologies.

### Viral and cellular RNA extraction and quantification

RNA from cells at different time points and conditions was extracted to determine gene expression or replicative intermediate production of HBV using the QIAshredder and RNeasy Mini Kit (Qiagen) according to the manufacturer’s protocol. cDNA was synthesized using High Capacity cDNA Reverse Transcription Kit provided by Applied Biosystems (Carlsbad, CA, USA) as described by the manufacturer’s protocol. qPCR quantification of ISGs, entry factors, PRRs, pgRNA, and sgRNA were performed using Platinum SYBR Green qPCR SuperMix-UDG w/ROX provided by Life Technologies on a ViiA™ 7 Real-Time PCR System instrument (Applied Biosystems) following the manufacturer’s protocol (Supplementary Table 3). Gene expression levels for NTCP, ASGPR, CYP2A6, HNMT, ABCB11, CYP2A13, GSTP1, and SLC22A1 were determined using Taqman Real-Time PCR assays (Life Technologies). The level of expression in each case was normalized to the housekeeping gene RPS11 or GAPDH.

### Immunofluorescence microscopy

All brightfield and immunofluorescence microscopy was performed using an EVOS FL Auto microscope (Life Technologies).

### Metabolite quantification by mass spectrometry

Conversion of Tacrine (5 µM) to 1-hydroxytacrine, Diclofenac (90 µM) to 4-hydroxydiclodenac, and Midazolam (5 µM) to 1-hydroxymidazolam was used to quantify the activities of CYP-1A2, CYP-2C9, and CYP3A4, respectively. A single cocktail of substrates was prepared at a 1000-fold concentration in DMSO and added to medium immediately before incubation. Microtissues were exposed to drugs by performing a wash followed by full medium change and incubated for 1 h under standard LiverChip culture conditions. Metabolites were quantified by mass spectrometry against quantitative standard curves by an independent contract research organization (Xenogesis Ltd, Nottingham UK). For N-acetyl-p-aminophenol (APAP) metabolite quantification, APAP was dissolved directly in cell culture medium to a final concentration of 1 mM. APAP metabolites formed after 6 or 24 h incubation were quantified in media samples using a quadrupole linear ion trap mass spectrometer (AB Sciex 4000 QTrap) coupled to a Dionex Ultimate 3000 HPLC system. Ten microliters of each sample was separated with a Phenomenex Kinetex 2.6u C18 100 A 100 × 2.10 mm column. A gradient consisting of 0.1% formic acid in water (mobile phase A) and acetonitrile (mobile phase B) was used with a flow rate of 300 μL/min. The column oven and auto-sampler were maintained at 40 and 4 °C, respectively. The mass spectrometer was operated using the multiple reaction-monitoring mode, and the analytes were detected and quantified using the most abundant transitions obtained during direct infusion of standards.

### CYP450 activity

CYP3A activity of 3D PHH cultures was determined using the P450-Glo^TM^ Assay (Promega) according to the manufacturer’s instructions.

### Electron microscopy

For transmission electron microscopic analysis, scaffolds were fixed overnight at 4 °C with 2.5% (*v*/*v*) glutaraldehyde in 0.1 M cacodylate buffer (pH 7.3) and post-fixed in 1% (*w*/*v*) osmium tetroxide in 0.1 M cacodylate buffer (pH 7.3) for 1 h at 4 °C. Scaffolds were then dehydrated through a graded ethanol series before infiltration with TAAB epoxy resin. Resin blocks were polymerized at 70 °C for 24 h. In some cases after resin infiltration, cells growing within the scaffold channels will become loose within the channel while keeping the 3D growth orientation and therefore could be embedded directly on the resin block. In other cases, the whole scaffold was embedded flat in the mold and then a razor blade was used to carefully trim around the scaffolding. Ultrathin sections (70–90 nm) were prepared using a Reichert-Jung Ultracut E ultramicrotome, mounted on 150 mesh copper grids and contrasted using uranyl acetate and lead citrate. Samples were examined on an FEI Tecnai 12 transmission microscope operated at 120 kV. Images were acquired with an AMT 16000M camera.

### Statistical analysis

Sample sizes were calculated based on an average expected effect size of 70% using alpha and beta levels of 0.05 and 0.5, respectively. Statistical significance was tested using two-way analysis of variance with Bonferroni post-test in Prism v5.0 (GraphPad). A value of *p* < 0.05 was considered significant.

### Ethical approval

All HBV serum samples used in this study were obtained from patients at St Mary’s hospital according to the ethical approval. Informed written consent was obtained from all patients through the Imperial College Hepatology Biobank and the Imperial College Tissue bank. The study was approved by all required ethics committees and regulatory authorities (REC reference 10/H0606/81, Hep-MD-15-003).

### Data availability

All relevant data are available from the authors upon request.

## Electronic supplementary material

Supplementary Information

Description of Additional Supplementary Files

Supplementary Movie 1

Supplementary Movie 2

Supplementary Movie 3

Supplementary Movie 4

Supplementary Movie 5

Supplementary Movie 6

Supplementary Movie 7
