# Supplementary material for: 3D microfluidic liver cultures as a physiological preclinical tool for hepatitis B virus infection
Source: Nat Commun. 2018 Feb 14;9:682. doi: 10.1038/s41467-018-02969-8 (PMC5813240; doi:10.1038/s41467-018-02969-8)
Supplement: Supplementary file 1 — Supplementary Information [file 41467_2018_2969_MOESM1_ESM.pdf]

3D microfluidic liver cultures as physiological preclinical tool for hepatitis B virus infection

Ortega-Prieto et al.

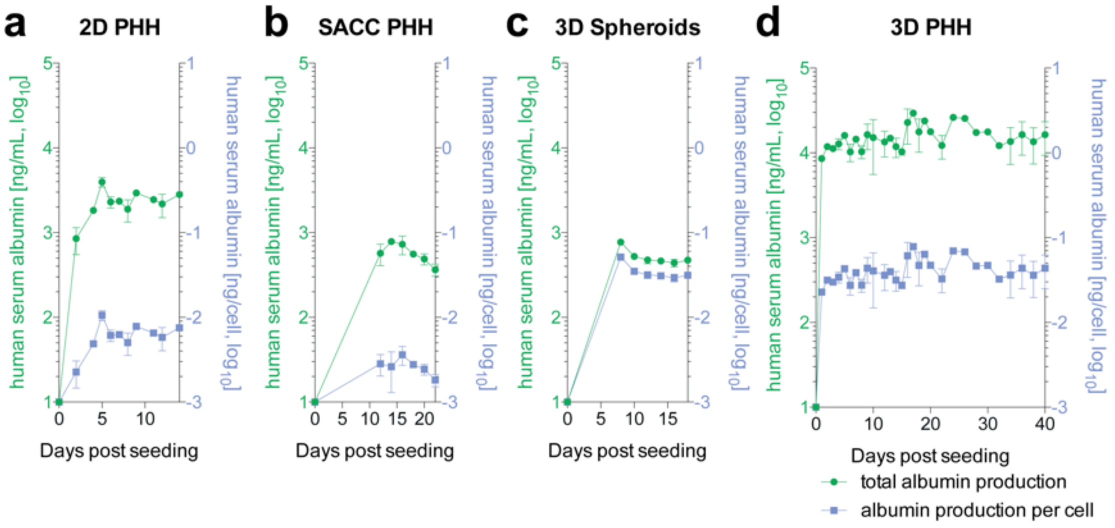

Supplementary figure 1. Longitudinal albumin secretion of different hepatocyte culture models. (**a-c**) Total and per cell albumin secretion kinetic of (**a**) 2D PHH cultures, (**b**) SACC PHH cultures, (**c**) 3D hepatic spheroid cultures and (**d**) 3D PHH cultures. Data shown are mean ± SD of six independent experiments.

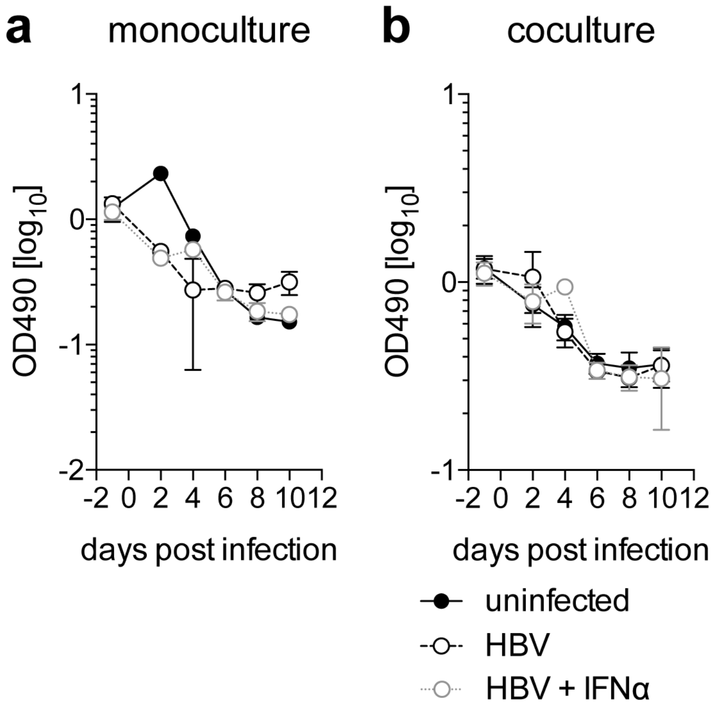

Supplementary figure 2. 3D PHH cultures are stable over time. Longitudinal release of lactate dehydrogenase (LDH) in (**a**) 3D PHH and (**b**) 3D PHH/KC co-cultures in the absence or presence of HBV infection with patient-derived HBV (100 GE/cell) and IFNα treatment (1000IU/mL). Data shown are mean ± SD of three independent experiments

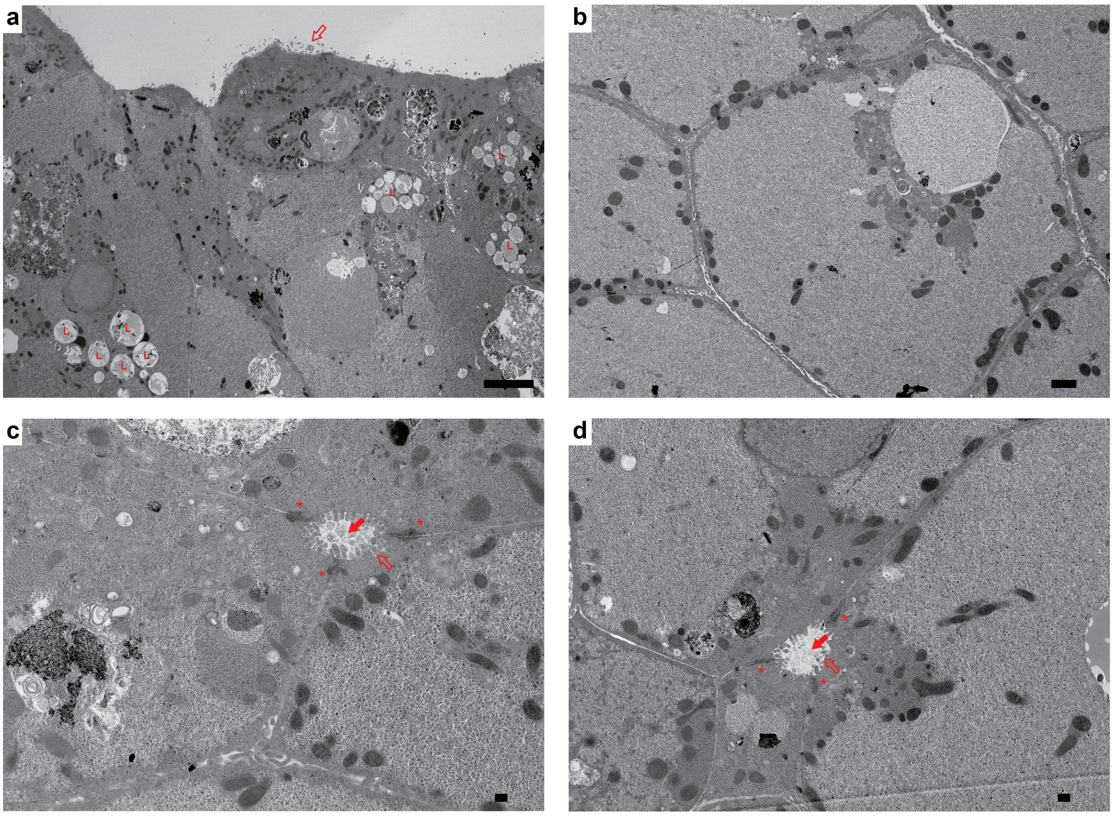

Supplementary figure 3. 3D PHH cultures adopt a human hepatic microarchitecture. (**a-d**) Transmission electron micrographs of PHH grown in 3D cultures for 20 days showing (**a**) basolateral growth areas with hepatic microvilli (open arrows) and lipid droplet formation (L), (**b**) cuboidal cellular structure of PHH and (**c, d**) bile canaliculi (closed arrows) and tight junctions (asterisk). Scale bar: 1μm

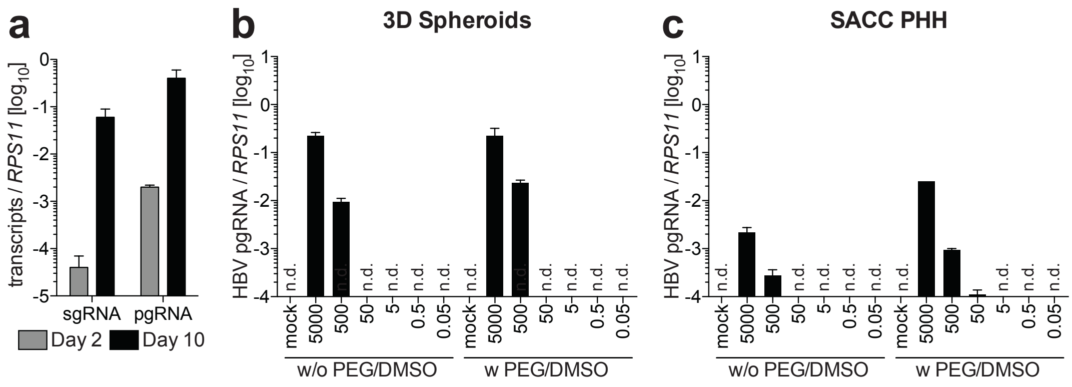

Supplementary figure 4. HBV infection of hepatocyte culture results in accumulation of HBV replication intermediates. (**a**) Intracellular HBV subgenomic (sg) and pregenomic (pg) RNA in 3D PHH cultures 2 days and 10 days post-infection with 100 GE/cell patient-derived HBV. (**b**, **c**) Intracellular pgRNA accumulation after 12 days of infection of (**b**) 3D spheroid cultures and (**c**) SACC PHH cultures with the indicated MOI of sucrose-purified HepDE19-derived HBV. Data shown are mean ± SD of three independent experiments.

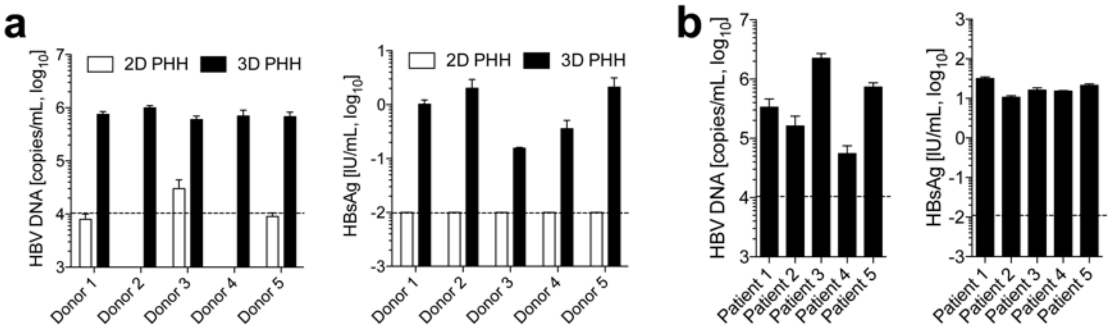

Supplementary figure 5. 3D PHH cultures are susceptible to HBV infection independent of hepatocyte or HBV origin. (**a**) Susceptibility of five different PHH donors cultured in 2D and 3D to infection with patient-derived HBV (100 GE/cell) as determined by HBV DNA and HBsAg secretion 10 days after infection. (**b**) Ability of five different patient-derived HBV isolates (100 GE/cell) to establish infection in 3D PHH cultures as determined by cumulative HBV DNA and HBsAg secretion 10 days after infection. Data shown are mean ± SD of three independent experiments.

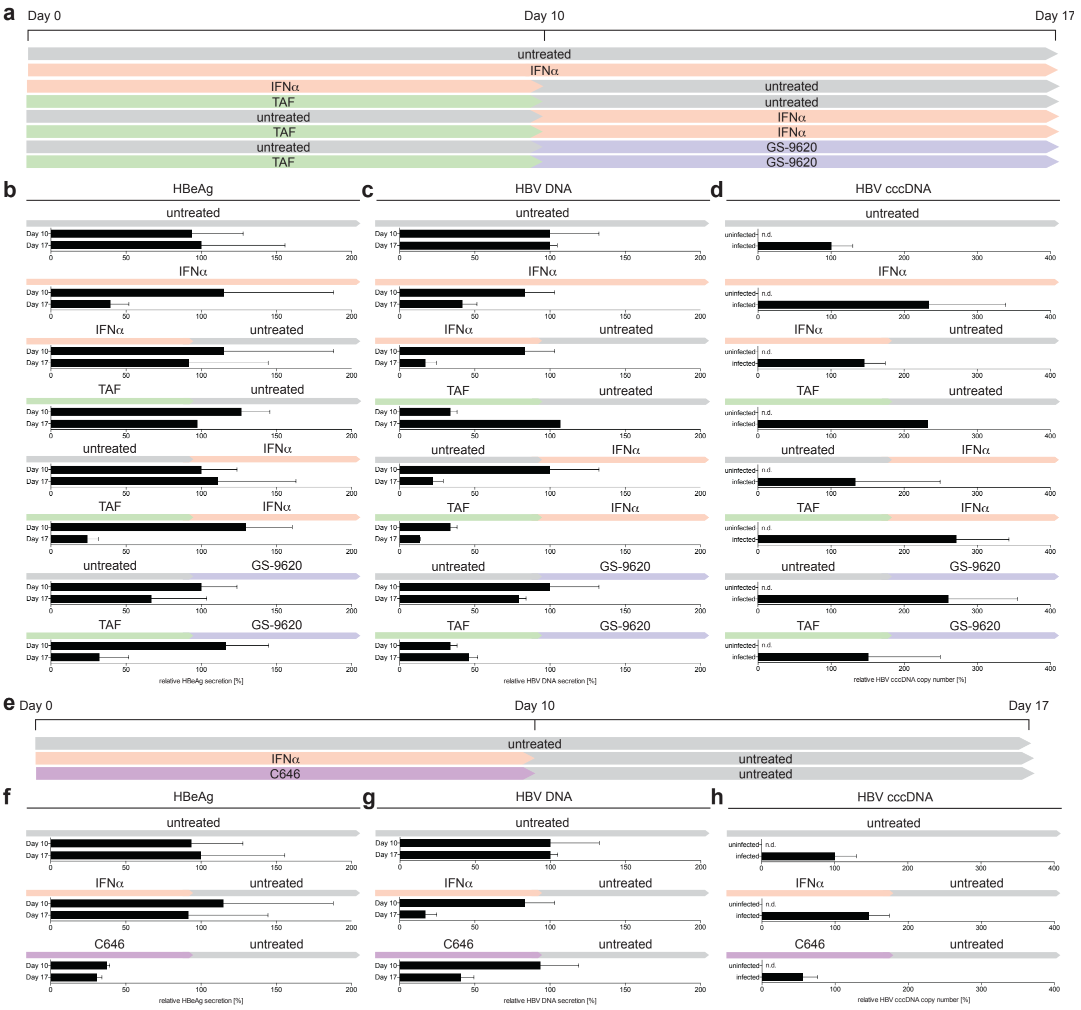

Supplementary figure 6. 3D PHH cultures can be utilized to evaluate antiviral treatment efficacies in subsequent treatment studies. (**a**) Experimental setup of the subsequent treatment of 3D PHH cultures infected with sucrose-purified HepDE19-derived HBV (500 GE/cell) with either 1000 IU/mL IFNα or 1μM TAF for 10 days followed by treatment with 1000IU/mL IFNα or 1μM GS-9620 for 7 days. (**b-d**) Evaluation of (**b**) HBeAg and (**c**) HBV DNA after 10 days (end of first treatment) and 17 days (end of second treatment) post-infection as well as (**d**) intracellular cccDNA at the final time-point. (**e**) Experimental setup for the comparison of IFNα and the p300 histone acetyltransferase inhibitor C646 in durably suppressing HBV replication by initiating treatment for 10 days followed by a 7-day drug-withdrawal period. (**f-h**) Evaluation of (**f**) HBeAg and (**g**) HBV DNA after the initial 10 days of treatment and

7 days post drug-withdrawal, as well as (**h**) intracellular cccDNA at the final time-point. Data shown are mean ± SD of three independent experiments.

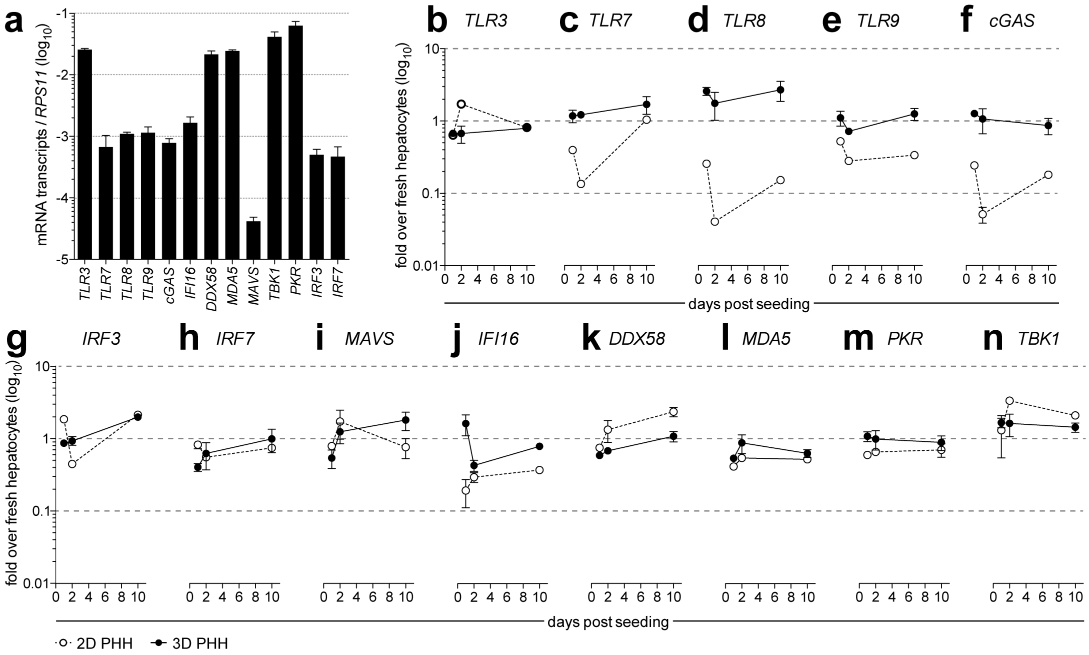

Supplementary figure 7. 3D PHH cultures stably maintain innate immune sensor and signaling mediator mRNA expression levels. (**a**) mRNA expression profile of freshly thawed PHH and (**b**) longitudinal fold change following culture in 2D or 3D of *TLR3, TLR7, TLR8, TLR9, cGAS, IRF3, IRF7, MAVS, IFI16, DDX58/RIG-I, MDA5, PKR*, and *TBK1*. Data shown are the mean ± SEM of two PHH donors.

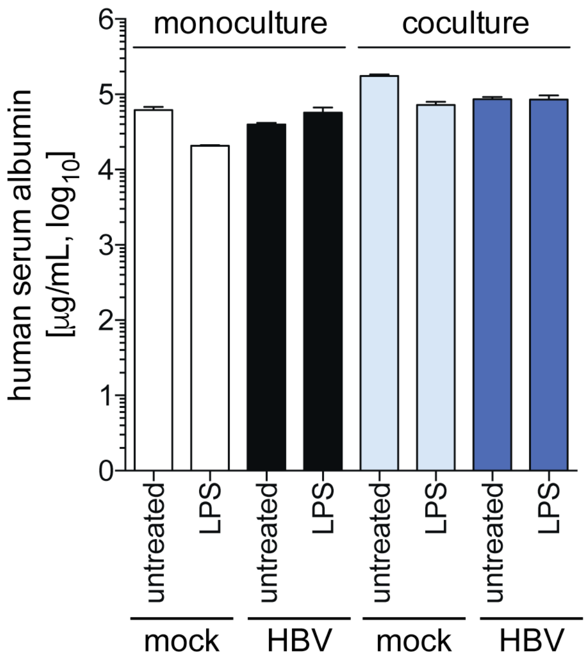

Supplementary figure 8. Albumin production is stable in 3D PHH and 3D PHH/KC co-cultures. HBV infection or treatment with 1μg/mL LPS does not affect albumin secretion at 10 days post-infection. Data shown are mean ± SD of three independent experiments.

Supplementary table 1. Primary cell donor information

Lot number	Gender	Age	Cell
HU1583	Female	58 years	Hepatocyte
HU8179	Female	55 years	Hepatocyte
HU1624	Female	72 years	Hepatocyte
HU4091	Not available	Not available	Hepatocyte
HUM4019	Male	1 year	Hepatocyte
HUM4025	Female	39 years	Hepatocyte
QHU0028	Female	1 year	Hepatocyte
QHU0032	Female	50 years	Hepatocyte
HK8180	Female	62 years	Kupffer cells
HK8226	Male	61 years	Kupffer cells

Supplementary table 2. HBV-infected patients

HBV DNA (copies/mL)	HBsAg (IU/mL)	HBeAg status	Treatment history
1270	27.6	Negative	Naïve
1144	4442	Negative	Naïve
9717	297	Negative	Naïve
23842	1837	Negative	Naïve
14985	0.38	Negative	TVR
27871	4440	Negative	Naïve
12390	0.11	Negative	Naïve
10429	2618	Negative	Naïve
21273	2490	Negative	TVR
25356	647	Negative	TVR
634436	1495	Negative	Naive

Supplementary table 3. Primers used for gene expression analysis

Gene	Forward (5' – 3')	Reverse (5' – 3')
IL28B (IFNl3)	TAAGAGGGCCAAAGATGCCTT	CTGGTCCAAGACATCCCCC
IFITM3 (1-8U)	CTGGGCTTCATAGCATTCGCCT	AGATGTTCAGGCACTTGGCGGT
IFI27	CGTCCTCCATAGCAGCCAAGAT	ACCCAATGGAGCCCAGGATGAA
OASL	GCACGAGACATCCACTTGACA	GGGAGAAGATCCCATATTTGGCT
IFI44L	GAGCACAGAAATAGGCTTCTAGC	TGGTATCAGACCCCACTACGG
RPS11	GCCGAGACTATCTGCACTAC	ATGTCCAGCCTCAGAACTTC
IFNb	GTCAGAGTGGAAATCCTAAG	ACAGCATCTGCTGGTTGAAG
IFNa1	CTGAATGACTTGGAAGCCTG	ATTTCTGCTCTGACAACCTC
MX1 (IFI78)	TCCCACCCTCTATTACTGAATGG	GGGAAGGGCAACTCCTGAC
IL29 (IFNl1)	GTGACTTTGGTGCTAGGCTTG	GCCTCAGGTCCCAATTCCC
IFIT1 (ISG56)	ACACCTGAAAGGCCAGAATG	GGTTTTCAGGGTCCACTTCA
CXCL10 (IP-10)	AGGAACCTCCAGTCTCAGCA	ATTTTGCTCCCCTCTGGTTT
TLR1	TTTGAAAATTGTGGGCACCTTACTG	AAGCAACATTGAGTTCTTGCAAAGC
TLR2	TGTGAACCTCCAGGCTCTG	GTCCATATTTCCCACTCTCAGG
TLR3	TCCCAAGCCTTCAACGACTG	TGGTGAAGGAGAGCTATCCACA
TLR7	TTACCTGGATGGAAACCAGCTAC	TCAAGGCTGAGAAGCTGTAAGCTA
TLR8	GAGAGCCGAGACAAAAACGTTC	TGTCGATGATGGCCAATCC
TLR9	TGGTGTTGAAGGACAGTTCTCTC	CACTCGGAGGTTTCCCAGC
DDX58	GGACGTGGCAAAACAAATCAG	GCAATGTCAATGCCTTCATCA
MDA5	CGGATATAAAGAATGTAACATTGTTATCCG	ATGAGCATACTCCTCTGGTTTCA
PKR	GGATTTGGCCAAGTTTTCAA	ATCAAATCCATCCCAACAG
cGAS	GGGATCCCGGCAGAAAAAGA	TTCAGTCTGAGCAGCAGGTG
IRF3	TACGTGAGGCATGTGCTGA	AGTGGGTGGCTGTTGGAAAT
IRF7	TGCAGAAGGTGGTGGGACA	TGCTATCCAGGGAAGACACA
MAVS	ATGCCGTTTGCTGAAGAC	CTAGTGCAGACGCCGCCG
IFI16	CCGTTCATGACCAGCATAGG	TCAGTCTTGGTTTCAACGTGGT
TBK1	GCAGTTTGTTTCTCTGTATGGC	AATGTTACCCCAATGCTCCA
IFI44	CGGTTGCACGAAAAGATCCTG	GTCAAGCAAAACTCCATTACGGA
CD81	TGTTCTTGAGCACTGAGGTGGTC	TGGTGGATGATGACGCCAAC
SCARB1	CGGATTTGGCAGATGACAGG	GGGGGAGACTCTTCACACATTCTAC
CLDN1	CACCTCATCGTCTTCCAAGCAC	CCTGGGAGTGATAGCAATCTTTG
OCLN	CGGCAATGAAACAAAAGGCAG	GGCTATGGTTATGGCTATGGCTAC
LDLR	TACAAGTGGGTCTGCGATGG	TGAAGTCCCCGGATTTGCAG
NPC1L1	CCACGAGAGGTCCACATTGG	GAAGAAGCAGATGGCCTCAGA
EGFR	GCTACGATTGGCTGAAGTAC	ATTGGGTGTAGAGAGACTGGA
pgRNA	GAGTGTGGATTCGCACTCC	GAGGCGAGGGAGTTCTTCT
sgRNA	TCACCAGCACCATGCAAC	AAGCCACCCAAGGCACAG
