# Supplementary material for: 3D microfluidic liver cultures as a physiological preclinical tool for hepatitis B virus infection
Source: Nat Commun. 2018 Feb 14;9:682. doi: 10.1038/s41467-018-02969-8 (PMC5813240; doi:10.1038/s41467-018-02969-8)
Supplement: Supplementary file 2 — Description of Additional Supplementary Files [file 41467_2018_2969_MOESM2_ESM.pdf]

Description of Additional Supplementary Files

File Name: Supplementary Movie 1
Description: **Albumin expression and morphology of 3D spheroid cultures in response to HBV infection.** Brightfield and immunofluorescence detection of albumin (green) and DAPI (blue) in 3D spheroid cultures 12 days following infection with the indicated MOI.

File Name: Supplementary Movie 2
Description: **Expression of CD81 in 3D PHH cultures.** Immunofluorescence detection via 3D reconstruction (left) and Z-stack (right) of CD81 in 3D PHH cultures 26 days post-seeding.

File Name: Supplementary Movie 3
Description: **Expression of GJB1 in 3D PHH cultures.** Immunofluorescence detection via 3D reconstruction (left) and Z-stack (right) of GJB1 in 3D PHH cultures 26 days post-seeding.

File Name: Supplementary Movie 4
Description: **Expression of ITGB1 in 3D PHH cultures.** Immunofluorescence detection via 3D reconstruction (left) and Z-stack (right) of ITGB1 in 3D PHH cultures 26 days post-seeding.

File Name: Supplementary Movie 5
Description: **Expression of ZO-1 in 3D PHH cultures.** Immunofluorescence detection via 3D reconstruction (left) and Z-stack (right) of ZO-1 in 3D PHH cultures 26 days post-seeding.

File Name: Supplementary Movie 6
Description: **HBV infection in 3D PHH cultures.** Immunofluorescence detection via 3D reconstruction (left) and Z-stack (right) of HBcAg in 3D PHH cultures, either uninfected (top) or infected with patient-derived HBV (200 GE/cell) (bottom) at 22 days post-infection.

File Name: Supplementary Movie 7
Description: **HBcAg expression and morphology of 3D spheroid cultures in response to HBV infection.** Brightfield and immunofluorescence detection of HBcAg (red) and DAPI (blue) in 3D spheroid cultures 12 days following infection with the indicated MOI.
